# An Improved Timed Elastic Band (TEB) Algorithm of Autonomous Ground Vehicle (AGV) in Complex Environment

**DOI:** 10.3390/s21248312

**Published:** 2021-12-12

**Authors:** Jiafeng Wu, Xianghua Ma, Tongrui Peng, Haojie Wang

**Affiliations:** School of Electrical and Electronic Engineering, Shanghai Institute of Technology, Shanghai 201418, China; 196102105@mail.sit.edu.cn (J.W.); 206101104@mail.sit.edu.cn (H.W.)

**Keywords:** local path planning, the shortest distance constraint, TEB algorithm, AGV

## Abstract

In recent decades, the Timed Elastic Band (TEB) algorithm is widely used for the AGV local path panning because of its convenient and efficiency. However, it may make a local detour when encountering a curve turn and cause excessive energy consumption. To solve this problem, this paper proposed an improved TEB algorithm to make the AGV walk along the wall when turning, which shortens the planning time and saves energy. Experiments were implemented in the Rviz visualization tool platform of the robot operating system (ROS). Simulated experiment results reflect that an amount of 5% reduction in the planning time has been achieved and the velocity curve implies that the operation was relatively smooth. Practical experiment results demonstrate the effectiveness and feasibility of the proposed method that the robots can avoid obstacles smoothly in the unknown static and dynamic obstacle environment.

## 1. Introduction

The AGV is currently widely used in the production workshop environment, mainly to select the best transportation path. It is urgently required to be able to consider the unstructured, clustered, uncertain and dynamic environments with the presence of humans, vehicles, and even other autonomous devices [1] in local path planning. Therefore, to improve the transport efficiency of AGV in such environments, the most important issue is that the AGV must safely avoid both static and dynamic obstacles during the course of path planning. Local path planning methods for the complex workshop environment include the artificial potential field method (APF) [2], the genetic algorithm [3], the dynamic window method (DWA) [4], the neural network method [5], the fuzzy logic method [6], and other intelligent optimization algorithms. When the above planning algorithm solves path planning problems in complex environments such as concave, cornered wall, and narrow passages, the convergence speed or the ability to avoid local extremes are significantly reduced. Xu F [7] proposed to set virtual target points in the potential field environment in order to increase the external force to escape from the local minimum. Mai X et al. [8] improved the DWA trajectory evaluation function to avoid entering dense areas in advance. Wang X Y et al. [9] proposed that the potential field method is combined with the ant colony algorithm, and that the heuristic function is reconstructed by using the artificial potential field method to avoid the occurrence of local optimal solutions. Lee et al. [10] improved the selection operator to solve the problem of slow convergence of genetic algorithm, and proposed a fast genetic algorithm.

The Timed Elastic Band (TEB) algorithm [11], initially proposed by Rösmann, is developed by the typical EB algorithm [12], is an online collision avoidance method for multi-objective online trajectory optimization. Compared with other local path planning algorithms, the TEB algorithm can add or delete several constraints according to different needs in different settings, and has a high degree of flexibility. The multi-objective optimization of the TEB algorithm only depends on a few consecutive configurations, which leads to the sparse structure of the underlying optimization problem. Rösmann et al. [13] proposed that the G2o framework could be used to quickly and effectively solve the sparsity of the TEB algorithm based on the hyper-graph, which has improved the calculation speed. In addition, the AGV equipped with the TEB algorithm is unable to transit across obstacles in the complex and dynamic environment, which easily causes the AGV to fall into a local minimum. To deal with that problem, Rösmann et al. [14] and Rösmann et al. [15] proposed extensions of the TEB technique by using parallel trajectory planning in spatially distinctive topologies. However, these approaches only take into account the position of the obstacle and do not incorporate the potential collisions between the robots and the surrounding obstacles. Lan at al. [16] proposed a proactive timed elastic band (PTEB) technique for autonomous mobile robot navigation systems in dynamic social environments. The previous work about improving the operation effect of the TEB algorithm in the complex environment focused on avoiding obstacles. Furthermore, most of the relevant research only pursues the avoidance of local minimums and the smoothing of planned paths for AGVs in the complex environment. It lacks the constraint of considering the shortest local path, and the local path planned is not the optimal path. Therefore, the improved TEB algorithm mentioned above still has the problem that local detour is easy to occur due to the excessive steering when turning on a curve.

Based on the above analysis, the improved TEB algorithm is proposed to generate the shortest AGV path. The shortest distance constraint is introduced to the multi-objective optimization problem constraints for the TEB algorithm, and the consumption caused by the excessive turning is considered. Then, the improved algorithm proved that the AGV can walk along the wall and close to the edge of the curved path when turning, and can shorten the time of the AGV local path planning.

The layout of this paper is as follows. Section 2 explains improved local path planning method of TEB algorithm in details. Next, Section 3 presents the simulation experiments on ROS system for testing the proposed method, and the real robot experiment is presented from two scenes of unknown static obstacles and dynamic obstacles. Then, Section 4 provides relevant results analysis. Finally, the conclusion is given in Section 5.

## 2. Proposed TEB Algorithm

### 2.1. Timed Elastic Band Algorithm Model Construction

The TEB algorithm includes geometric constraints based on the AGV kinematics model in the multi-objective constraints. The differential AGV model [17] has the advantages of convenient movement, rapid turning, and wide application range. It is convenient for real-time control when path planning is carried out in the complex workshop environment, as shown in Equation (1).

(1) $$u\left(t\right)=\left[\begin{array}{l} V\left(t\right)\\ \omega \left(t\right) \end{array}\right]=\left[\begin{array}{cc} \frac{1}{2} & \frac{1}{2}\\ -\frac{1}{D} & \frac{1}{D} \end{array}\right]\left[\begin{array}{l} V_{L}\\ V_{R} \end{array}\right]$$

where, $V_{L}$ and $V_{R}$ are the speeds of the left and right wheels of the AGV rear axle, and D is the distance between the two wheels.

**Figure 1:**
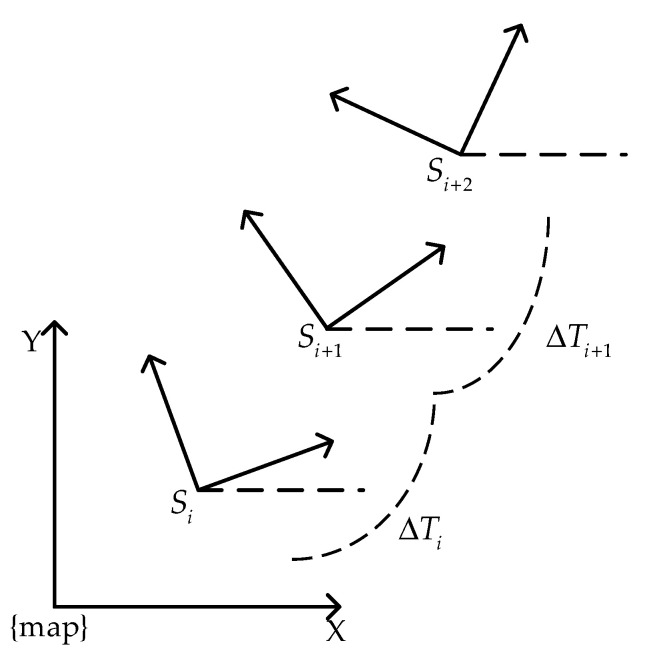
Pose and time interval of AGV in the world coordinate system.

The TEB algorithm increases the time interval information between the AGV pose sequences based on the EB algorithm, as shown in Equation (2), which lays the foundation for the robot to increase the speed and acceleration limits, the fastest path, the improved shortest distance, and other constraints. And the idea of graph optimization is introduced. The basic principle is to modify the global path with the trajectory model, transform the searched initial path into a trajectory sequence based on discrete-time, as shown in Figure 1. The pose states and corresponding time difference are solved by using the G2o [18] optimization package, and the optimal control quantity satisfying the constraint is obtained to control the movement of the AGV car in real-time, as shown in Equation (3).

(2) $$\left\{ \begin{array}{l} Q=\left\{S_{i}\right\},i=1,2,\ldots ,nn\in \mathbb{N}\\ \tau =\left\{\Delta T_{i}\right\},i=1,2,\ldots ,n-1 \end{array}\right.$$

(3) $$B:=\left(Q,\tau \right)=\left[S_{1},\Delta T_{1},S_{2},\Delta T_{2},\ldots ,\Delta T_{n-1},S_{n}\right]$$

where, $S_{i}$ is the pose at time i, and Q is the pose sequence; $\Delta T_{i}$ is the time interval between adjacent poses, τ is the time interval sequence; The pose sequence and the time interval sequence are combined into a trajectory sequence B.

The objective constraint functions of the TEB algorithm only depend on several contiguous pose states. This locality leads to the generation of the system sparse matrix. Therefore, the piecewise continuous differentiable function can be used as the penalty function to punish the violation of the constraint conditions, as shown in Equation (4).

(4) $$e_{\tau }\left(x,x_{r},\varepsilon ,S,n\right)\approx \left\{ \begin{array}{cc} {\left(\frac{x-\left(x_{r}-\varepsilon \right)}{S}\right)}^{n} & ,x>x_{r}-\varepsilon \\ 0 & ,x\leq x_{r}-\varepsilon \end{array}\right.$$

where, $x_{r}$ is the boundary value, S is the deformation factor, and n is the polynomial coefficient, which is usually valued at 2; ε is a small displacement (i.e., offset factor) near the boundary value.

**Figure 2:**
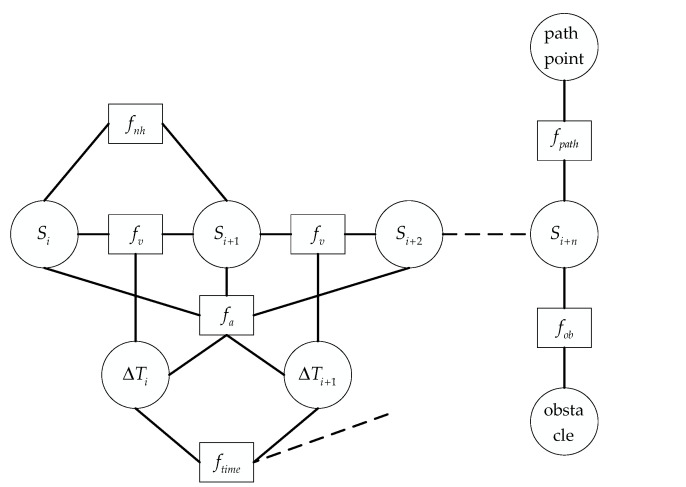
TEB hyper-graph structure.

The hyper-graph structure contained in the TEB constraint function is shown in Figure 2, and the TEB multi-objective optimization function is defined as:

(5) $$f\left(B\right)=\sum_{k}\gamma_{k}f_{k}\left(B\right)$$

where, $f_{k}\left(B\right)$ is each constraint function in Figure 2, and $\gamma_{k}$ is the weight corresponding to the constraint function.

$f_{v}=e_{\tau }\left(V_{i},V_{\max},\varepsilon ,S,n\right)$ is the speed constraint of the AGV, connecting the adjacent poses $S_{i}$ and $S_{i+1}$. $f_{a}=e_{\tau }\left(a_{i},a_{\max},\varepsilon ,S,n\right)$ is the acceleration constraint of the AGV, which forms an edge with $S_{i},S_{i+1}$ and $\Delta T_{i},\Delta T_{i+1}$. $f_{ob}=e_{\tau }\left(-d_{\min,j},-r_{o_{\min}},\varepsilon ,S,n\right)$ is the obstacle constraint caused by the smallest obstacle from $S_{i+n}$ in the current pose. $f_{path}=e_{\tau }\left(d_{\min,j},r_{p_{\max}},\varepsilon ,S,n\right)$ is the trajectory constraint generated by the nearest target waypoint. $f_{time}={\left(\sum_{i=1}^{n}\Delta T_{i}\right)}^{2}$ is the time constraint of each time interval, in the form of summing each time interval. $f_{nh}$ is the geometric constraint of AGV from pose $S_{i}$ to pose $S_{i+1}$, which is equivalent to the nonholonomic kinematics constraint of the differential AGV. The nonholonomic kinematics AGV is similar to the structure of the vehicle. The direction angle is controlled by the left and right differential wheels, which is difficult to meet the driving in any direction. And the movement of its two adjacent spatial poses $S_{i}$ and $S_{i+1}$ can be approximated as an arc motion with constant curvature. The angle between the two adjacent pose points and the direction of motion is equal.

### 2.2. AGV Local Path Planning Based on Improved TEB Algorithm

Similar to the task in a complex workshop environment. We define the path planning problem considered in this paper in general. During the movement of the AGV in the complex dynamic environment, the traditional TEB algorithm will slow down when encountering a curve, and then accelerate from the corner to the subsequent position. There will have the problems that local detour occurs easily when turning. In order to improve the efficiency of the TEB algorithm in real-time searching for the optimal path, and the quality of path planning, and combined with the practical issues such as AGV path planning safety and low power consumption. This paper uses the idea of the heuristic function of the A^*^ algorithm for reference, the traditional TEB algorithm is improved as follows: 

#### 2.2.1. A^*^ Algorithm Is Fused to Introduce the Shortest Distance Constraint

The A^*^ algorithm is a heuristic algorithm that considers the actual cost and the estimated cost. The evaluation function is used to estimate the total length of the path. The general cost estimation function is expressed as follows:

(6) f(n)=g(n)+h(n)

where, g(n) represents the actual cost from the initial point to the current node, h(n) represents the estimated cost from the current node to the target node, and the calculation equation for h(n) is as follows:

(7) $$h\left(n\right)=\sqrt{{\left(x_{G}-x_{n}\right)}^{2}+{\left(y_{G}-y_{n}\right)}^{2}}$$

where, $\left(x_{n},y_{n}\right)$ represents the horizontal and vertical coordinates of the map of the current point, and $\left(x_{G},y_{G}\right)$ represents the horizontal and vertical coordinates of the map of the target point.

In order to solve the problem of local detours when turning in TEB path planning, under the inspiration of the Equation (7), it is proposed to introduce the shortest distance constraint to make the local path close to the path edge, and improve the efficiency of AGV local path planning. TEB local path planning first plans a global path through the A* algorithm. In the initialization phase, a section of the global path is intercepted to obtain the initial local path, and the initial path is transformed into a trajectory composed of pose sequence and time sequence; In the iterative solution cycle stage, each iteration is carried out according to the number of sampling points n. In each iteration, a new pose is inserted or the previously processed pose is deleted to maintain the locally optimized trajectory length unchanged, and the obstacle information in the cost map is updated in actual time.

**Figure 3:**
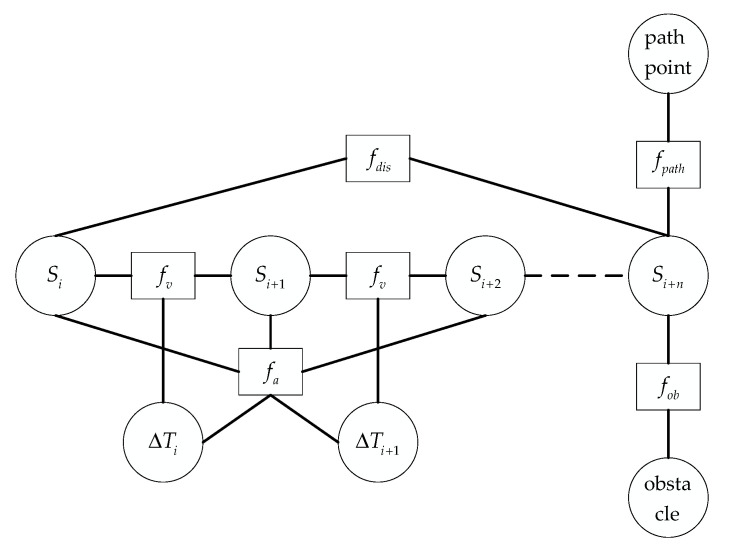
The improved hyper-graph.

Add the Euclidean distance between the current pose point $S_{i}$ and the pose point $S_{i+n}$ with an interval of n as the shortest distance constraint, and add the shortest distance constraint edge to the hyper-graph to construct a new hyper-graph, as shown in Figure 3. The shortest distance penalty function $f_{dis}$ is connected to the two pose vertices $S_{i}$ and $S_{i+n}$. The shortest distance constraint function can be expressed as follows:

(8) $$f_{dis}\left(S_{i},S_{i+n}\right)={\left(x_{i+n}-x_{i}\right)}^{2}+{\left(y_{i+n}-y_{i}\right)}^{2}$$

The total cost function after the improved TEB algorithm is composed of the weighted sum of the penalty and objective functions in Section 2.1.

(9) $$\begin{array}{ll} f\left(B\right)=\sum_{k}\gamma_{k}f_{k}\left(B\right)= & \gamma_{ob}\cdot f_{ob}\left(B\right)+\gamma_{path}\cdot f_{path}\left(B\right)+\\ & \gamma_{v}\cdot f_{v}\left(B\right)+\gamma_{a}\cdot f_{a}\left(B\right)+\\ & \gamma_{time}\cdot f_{time}+r_{dis}\cdot f_{dis} \end{array}$$

(10) $$B^{*}=\underset{B}{\min}f\left(B\right)$$

The optimization problem constitutes a nonlinear least squares problem, which is solved by the Levenberg-Marquardt algorithm. The weights $\gamma_{k}$ are chosen by a trial and error approach such the resulting trajectory complies the needs of the complex workshop environment. When the AGV is transported in a complex environment, unknown static obstacles and unknown dynamic obstacles need to be considered. Obviously, the shortest distance constraint weight for handling unknown dynamic obstacles is higher than that for unknown static obstacles.

#### 2.2.2. Implementation of AGV Local Path Planning Based on Improved TEB Algorithm

**Figure 4:**
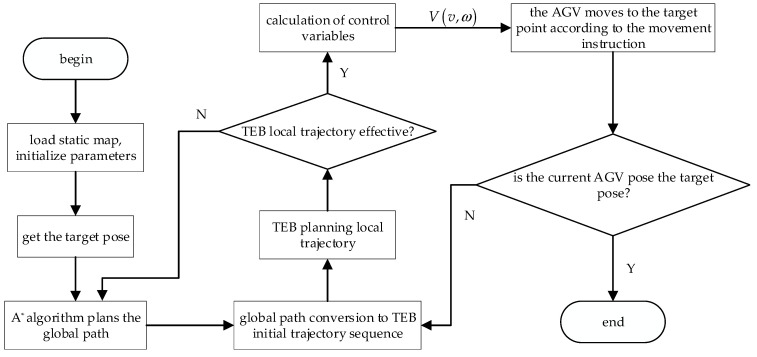
Path planning flow chart based on A* algorithm and improved TEB algorithm.

When the trajectory points optimized by TEB are located between obstacles or pass through obstacles, it makes up an invalid trajectory. When this happens, the A^*^ algorithm is enabled to re-plan the global path between the robot’s current pose and the target point, and then TEB parallel trajectory optimization of the real-time topology is carried out, as shown in Figure 4.

## 3. Simulation Experiment and Real Robot Experiment

### 3.1. Simulation Experiment

The simulation experiment was conducted on the Rviz visualization platform to demonstrate the capability of the proposed TEB algorithm. A four-wheel differential AGV was used those two rear axle wheels are differential driving wheels, and the others are the follower wheel. The steering is controlled by the speed difference of the driving wheels. 

Table 1 presents several key parameters of the simulated experiment.

### 3.2. Real Robot Experiment

The Turtlebot2 mobile robot equipped with the Kinect V1 camera and R2000 lidar was tested in a closed multi-obstacle scene. The experiments were conducted in an unknown environment with static or dynamic obstacles to verify the feasibility of the proposed method. The global path generated by the global path planner was used as the reference path in the robot navigation, and the proposed TEB algorithm optimizes the local path to control the AGV’s movement in real time.

## 4. Results Analysis

### 4.1. Simulated Experiment Results Analysis

**Figure 5:**
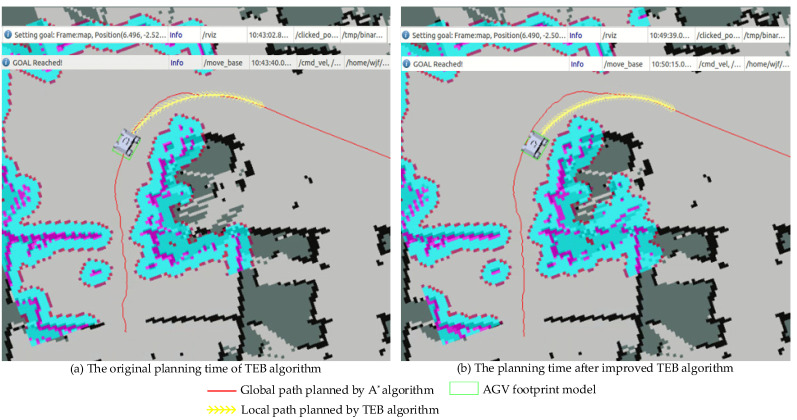
Time comparison of TEB algorithm planning before and after improvement.

Figure 5 presents the planning time for the traditional TEB algorithm and the proposed TEB algorithm for the AGV local path planning in the complex environment.

The yellow line denotes the local path planned by the TEB algorithm: Figure 5a presents the traditional TEB algorithm and Figure 5b presents the proposed TEB algorithm. Setting the same starting point, and it can be obtained from Table 2: The time for the traditional TEB algorithm was 37.2 s and the time for the proposed TEB algorithm was 36 s. It is clearly that the proposed TEB algorithm can reduce the planning time of the AGV by 5% on the basis of stable operation when compared to the traditional TEB algorithm and it contributes to improve the efficiency of path planning in the complex workshop environment. In addition, the planned local path is along the edge of the curve to avoid energy loss caused by over-steer.

**Figure 6:**
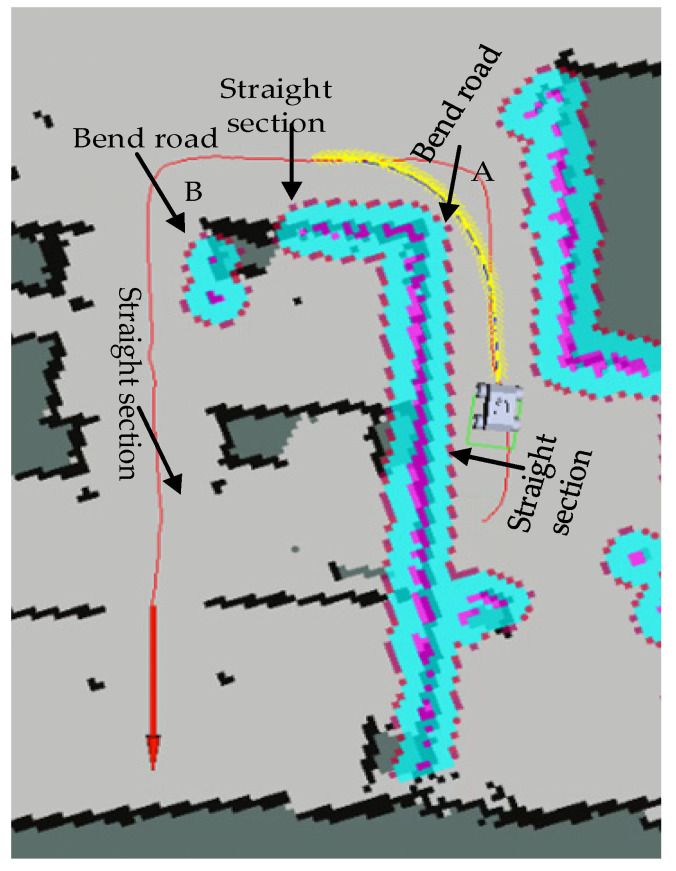
U-shaped path scenario for AGV operation.

In order to verify the smoothness of the AGV running linear velocity of the proposed TEB algorithm, Figure 6 presents the U-shaped path scene of the AGV running. Among them, the red line is the planned global path, and the yellow line is the real-time local path of the AGV. The starting point is at the bottom right, and the target pose is indicated by a red arrow. The U-shaped path contains a total of 3 straight line segments: starting point to point A, point A to point B, and point B to the target point, and it contains 2 curves.

**Figure 7:**
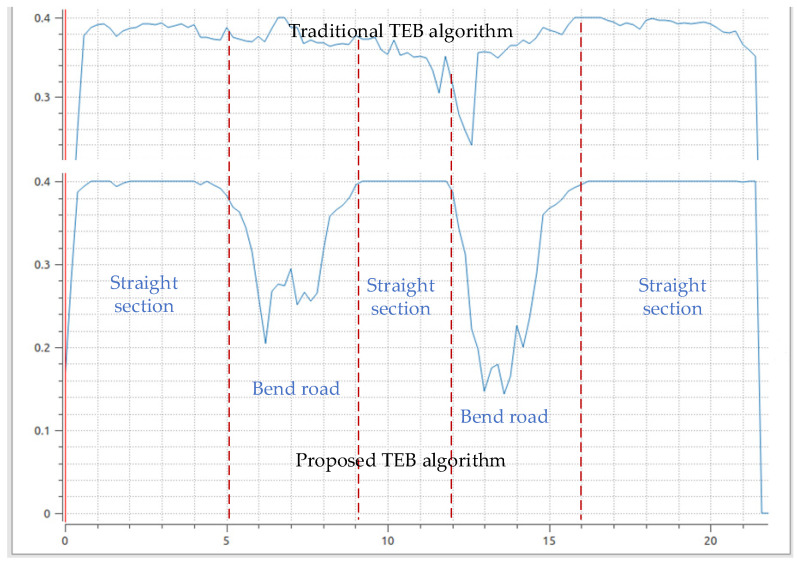
AGV speed output curve of the traditional TEB algorithm and the proposed TEB algorithm.

It can be seen from the Figure 7 that the speed curve of the proposed TEB algorithm is smoother, so that the AGV will not produce overshoot and oscillation in the actual scene. Therefore, the AGV is more stable when transporting goods, will not cause the goods to fall, and improve the efficiency of transportation. The proposed TEB algorithm has an obvious speed change process when encountering a curve. However, the traditional TEB algorithm does not significantly reduce the speed when the AGV encounters the first turn, which may cause some safety problems when the AGV is transporting goods.

### 4.2. Practical Experiment Results Analysis

**Figure 8:**
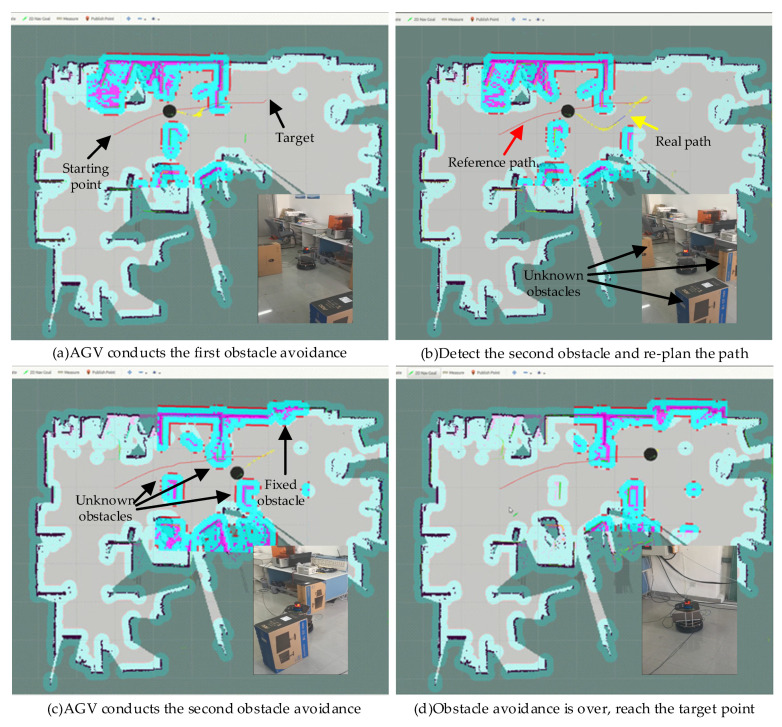
The process of AGV movement in the environment with unknown static obstacles.

Figure 8 shows the position of the turtlebot2 navigation visualized in ROS Rviz and the corresponding real turtlebot2 position in the environment with unknown static obstacles such as boxes, where the red line represents the reference path of the global plan. The AGV moves according to the local path planned by the TEB algorithm in real time, that is, the yellow line in the figure, and the cyan lines are the expansion range of the obstacles.

In this experiment, the shortest distance constraint weight is designed to be 30. To reflect the characteristic of “unknown”, the original scenario contains no obstacles. During the navigation of turtlebot2, there are multiple boxes as the unknown static obstacles. The tuetlebot2 has no prior knowledge of the obstacles and can only perceive the obstacles in real time. The spatial distribution of these obstacles is set to significantly intercepts the trajectory of the AGV towards the target. Specifically, Figure 8a,b show the starting position, the target position, and the real local path. Figure 8b–d show the movements of turtlebot2 avoiding the unknown static obstacles and the turtlebot2 successfully reaches the target. In Figure 8b, when an unknown static obstacle is encountered, the local path will be re-planned in real time, and turtlebot2 will follow the local path to move. The AGV moves flexibly between the multiple boxes, which proves that the improved method can navigate the AGV in the unknown environment.

**Figure 9:**
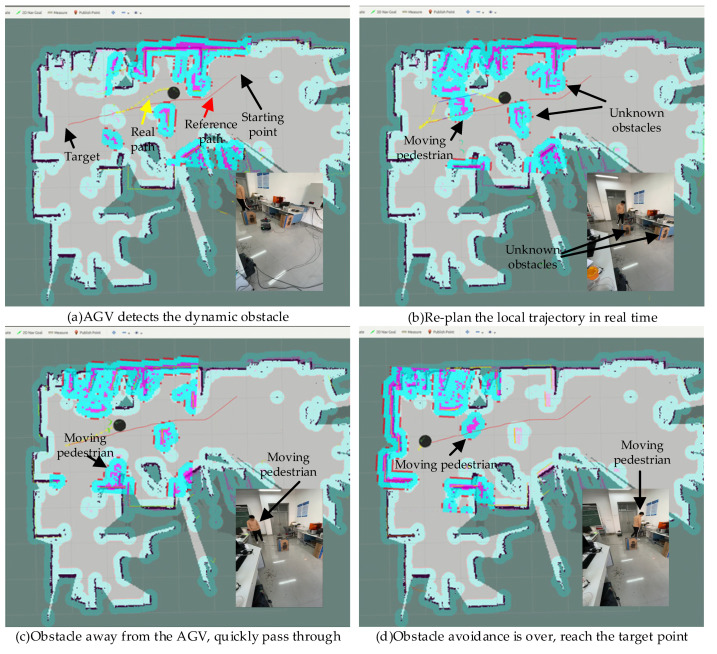
The process of AGV movement in the environment with the unknown dynamic obstacle.

Figure 9 shows the position of the turtlebot2 navigation visualized in ROS Rviz and the corresponding real turtlebot2 position in the experiment with the unknown dynamic obstacle. A pedestrian walks randomly in the scenario who significantly intercepts the trajectory of the robot towards the target once.

In this experiment, the shortest distance constraint weight is designed to be 50. Specifically, Figure 9a shows the starting position, the target position, the real local path and the moving pedestrian. Figure 9b–d show the movements of turtlebot2 avoiding the moving pedestrian. When a pedestrian hinders the AGV motion, the AGV will stop in place and re-plan the local path in real time. When the pedestrian is far from the AGV, the AGV smoothly and quickly bypasses unknown dynamic obstacles. It can be seen that the AGV avoids the moving pedestrian, which proves that the improved method can navigate the AGV in the simple unknown dynamic environment.

## 5. Conclusions

This paper proposed an improved TEB algorithm for the AGV local path planning in the complex environment. The proposed TEB algorithm can reduce the planning time of the AGV by 5%, and has the smooth speed curve in the straight section without overshoot and oscillation. In addition, the proposed TEB algorithm has an obvious speed change process when encountering a curve, and the planned local path is along the edge of the curve to avoid energy loss caused by over-steer. Both simulation and practical results reveal that the proposed method can find an effective and feasible path for the AGV to avoid obstacles smoothly in both unknown static and dynamic obstacle environment.

## Figures and Tables

**Table 1:** Parameter configuration of the simulated experiment.

Constraint Parameters	Values
Maximum X linear velocity (m/s)	0.4
Maximum backward linear velocity (m/s)	0.2
Maximum angular velocity (rad/s)	0.3
Maximum X linear acceleration (m/s^2^)	0.5
Maximum angular acceleration (rad/s^2^)	0.5
Obstruction expansion radius (m)	0.6
Minimum distance to obstacle (m)	0.25
the weight of shortest distance constraint	30

**Table 2:** Time comparison after improving TEB algorithm.

	Starting Point	Starting Time	End Time	Total Time Spent
Traditional TEB algorithm planning time	(6.496,−2.52)	10:43:02.8	10:43:40.0	37.2s
Improved TEB algorithm planning time	(6.490,−2.50)	10:49:39.0	10:50:15.0	36s

## Data Availability

Not applicable.
